# Silent Renal Autodestruction: Incidental Discovery of End-Stage Genitourinary Tuberculosis (Putty Kidney) 14 Years After Treatment for Pulmonary Tuberculosis With Multisystem Sequelae

**DOI:** 10.7759/cureus.113227

**Published:** 2026-07-23

**Authors:** Manasi Kurale, Swanand Chaudhary, Rohit Nimje

**Affiliations:** 1 Department of Surgery, N.K.P. Salve Institute of Medical Sciences and Research Centre and Lata Mangeshkar Hospital, Nagpur, IND; 2 Department of Urology, N.K.P. Salve Institute of Medical Sciences and Research Centre and Lata Mangeshkar Hospital, Nagpur, IND

**Keywords:** autonephrectomy, extrapulmonary tuberculosis, genitourinary tuberculosis, incidental renal calcification, putty kidney, renal tuberculosis, silent kidney

## Abstract

Genitourinary tuberculosis (GUTB) is a prevalent yet frequently underdiagnosed form of extrapulmonary tuberculosis (TB), owing to its characteristically indolent and asymptomatic clinical course. In its most advanced form, renal TB culminates in complete destruction and dystrophic calcification of the renal parenchyma, a condition known as "putty kidney" or autonephrectomy. This entity can develop silently over many years, even in patients who have completed antitubercular therapy for pulmonary disease.

A 44-year-old woman with a remote history of pulmonary TB treated 14 years prior presented to the gynecology outpatient department for the evaluation of menstrual irregularities. She denied all urinary symptoms. Incidental ultrasonography revealed gross hydronephrosis and calcification of the left kidney. Contrast-enhanced computed tomography (CT) of the abdomen demonstrated a calcified, nonexcreting left kidney consistent with a putty kidney, accompanied by calcified hepatic and splenic granulomas and calcified mesenteric lymph nodes. High-resolution CT of the thorax identified calcified pulmonary nodules consistent with healed pulmonary TB. Magnetic resonance imaging of the spine revealed lytic lesions at L4-L5 suggestive of healed spinal TB. A diethylenetriamine pentaacetic acid (DTPA) renal scan confirmed absent left renal function. The patient underwent open left simple nephrectomy. Histopathological examination demonstrated granulomatous inflammation with multinucleated giant cells and caseous necrosis, confirming renal TB. The postoperative course was uneventful.

Renal TB is characterized by slow, progressive hematogenous dissemination of *Mycobacterium tuberculosis*, leading to granuloma formation, fibrosis, and ultimately parenchymal calcification. Renal TB may become clinically apparent years after treated pulmonary TB. The underlying mechanism may involve reactivation of dormant bacilli or progression of previously established latent genitourinary infection, and the exact mechanism cannot be determined in this case. The absence of urinary symptoms can result in years of undetected progression, rendering incidental imaging an important diagnostic pathway. Nephrectomy is indicated for a nonfunctioning tuberculous kidney to prevent complications such as recurrent infection, persistent pain, uncontrolled hypertension, or when malignancy cannot be excluded.

This case illustrates that end-stage GUTB may remain clinically silent for years and be detected incidentally despite previous treatment for pulmonary TB. Long-term clinical vigilance should be maintained in patients with previous TB, particularly when new symptoms or suspicious imaging findings develop. Routine imaging surveillance for all patients is not established by current evidence.

## Introduction

Tuberculosis (TB) remains a major global health burden and continues to be one of the leading causes of death from infectious disease worldwide. Extrapulmonary TB accounts for a substantial proportion of cases, and genitourinary tuberculosis (GUTB) represents one of the most common forms, particularly in endemic regions such as India [1,2]. GUTB typically arises from hematogenous dissemination of *Mycobacterium tuberculosis* from a primary pulmonary focus, often years after the initial infection, reflecting its indolent and latent nature [2].

Renal involvement is the most frequent manifestation of GUTB, and the disease often progresses silently, with minimal or nonspecific clinical symptoms despite significant parenchymal destruction [3]. The pathogenesis involves granuloma formation, caseous necrosis, cavitation, and progressive fibrosis, ultimately leading to strictures, obstruction, and loss of renal function [4]. Over time, chronic inflammation and dystrophic calcification may replace the entire renal parenchyma, producing the characteristic radiological appearance known as a “putty kidney,” which represents an end-stage, nonfunctioning, autonephrectomized kidney [4].

The term “putty kidney” describes dense, homogeneous calcification within a shrunken kidney caused by longstanding TB and is now rarely encountered because of earlier diagnosis and treatment. However, delayed or missed diagnosis remains a significant clinical challenge, as GUTB can mimic other urological conditions and may remain clinically occult for years [5]. In many cases, patients present late with complications such as obstructive uropathy, hydronephrosis, or chronic kidney disease, sometimes discovered incidentally during imaging performed for unrelated conditions [6].

Renal TB may become clinically apparent years after successful treatment of pulmonary TB. This delayed presentation may result from reactivation of dormant bacilli or progression of previously established latent genitourinary infection; however, the exact mechanism cannot usually be determined in an individual patient. Consequently, clinicians should maintain awareness of the possibility of delayed genitourinary involvement in patients with a history of TB who develop compatible clinical or radiological findings. The occurrence of silent renal autodestruction culminating in a putty kidney years after treated pulmonary TB is rare and underscores the insidious progression of the disease.

Here, we report a case of incidental discovery of end-stage GUTB presenting as a putty kidney 14 years after treated pulmonary TB, associated with multisystem sequelae. This case emphasizes the importance of maintaining clinical suspicion and performing appropriate imaging when patients with a history of TB present with suggestive clinical or radiological findings.

## Case presentation

A 44-year-old woman presented to the gynecology outpatient clinic with complaints of irregular menstruation. She did not report any urinary symptoms such as flank pain, hematuria, dysuria, fever, or weight loss. Her medical history was significant for pulmonary TB 14 years earlier, for which she had completed a full one-year course of antitubercular therapy.

**Figure 1:**
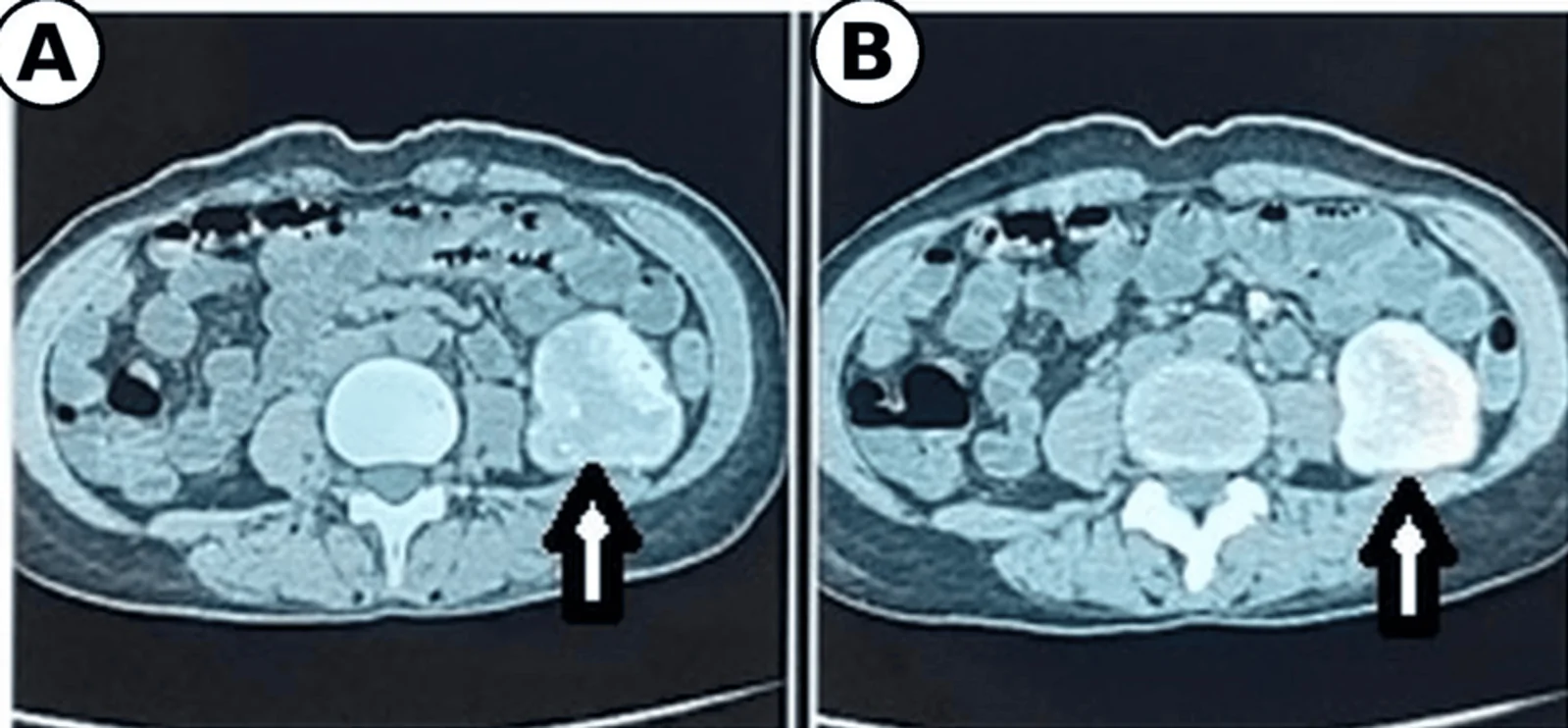
Contrast-enhanced CT of abdomen and pelvis showing a calcified left kidney (solid white arrows) Axial contrast-enhanced CT image (A) showing a calcified left kidney and (B) at an adjacent level, again showing the calcified left kidney with associated arrow marking CT: computed tomography

A routine ultrasonographic examination incidentally revealed marked hydronephrosis with calcification in the left kidney. Laboratory tests indicated normal renal function. A contrast-enhanced computed tomography (CT) scan of the abdomen demonstrated a heavily calcified, nonfunctioning left kidney, consistent with a putty kidney (Figure 1).

**Figure 2:**
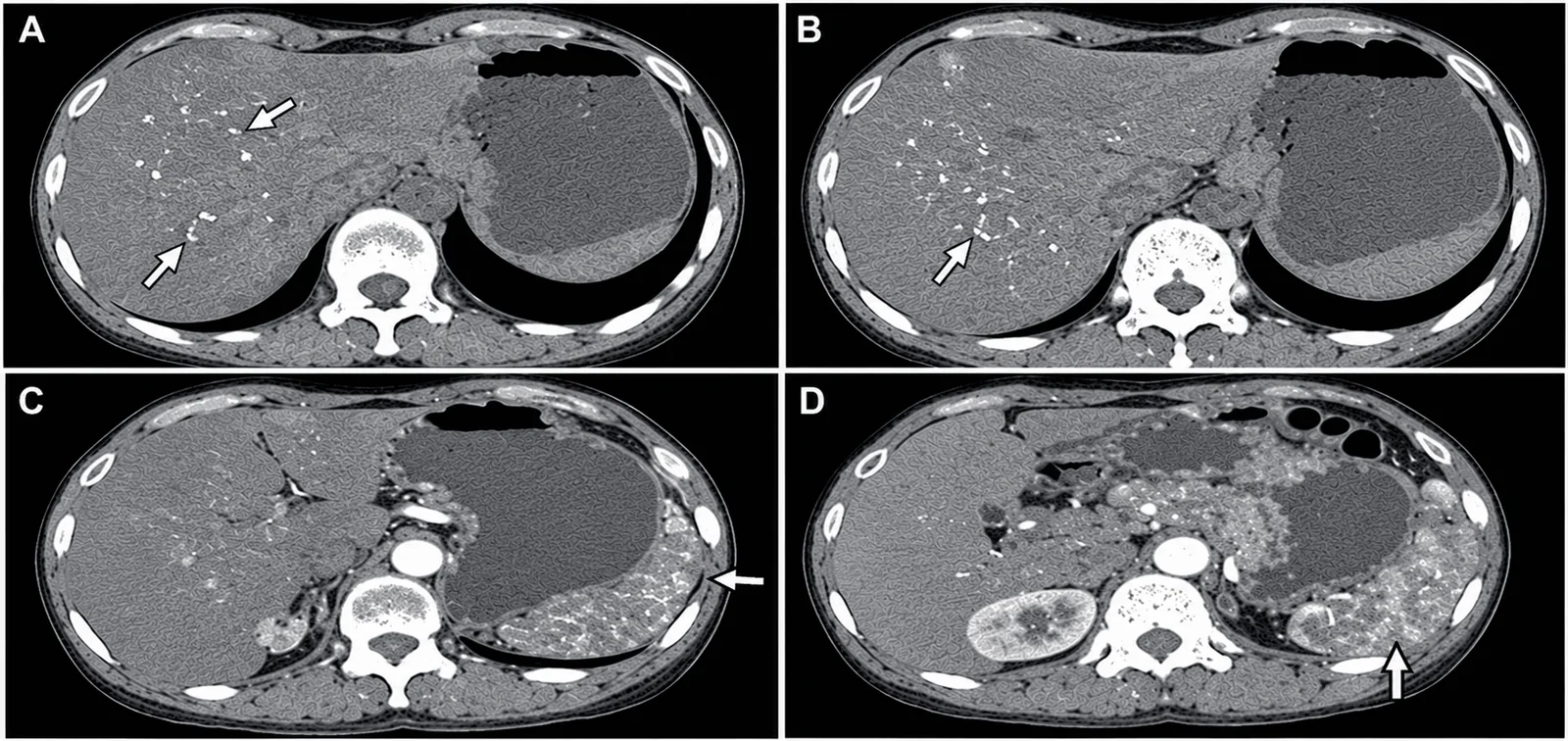
Contrast-enhanced CT scan of the abdomen and pelvis showing multiple calcified granulomas in the (A,B) liver (solid arrows) and (C,D) spleen (solid arrows)

Additionally, multiple calcified granulomas were noted in the liver and spleen, along with calcified mesenteric lymph nodes (Figure 2).

**Figure 3:**
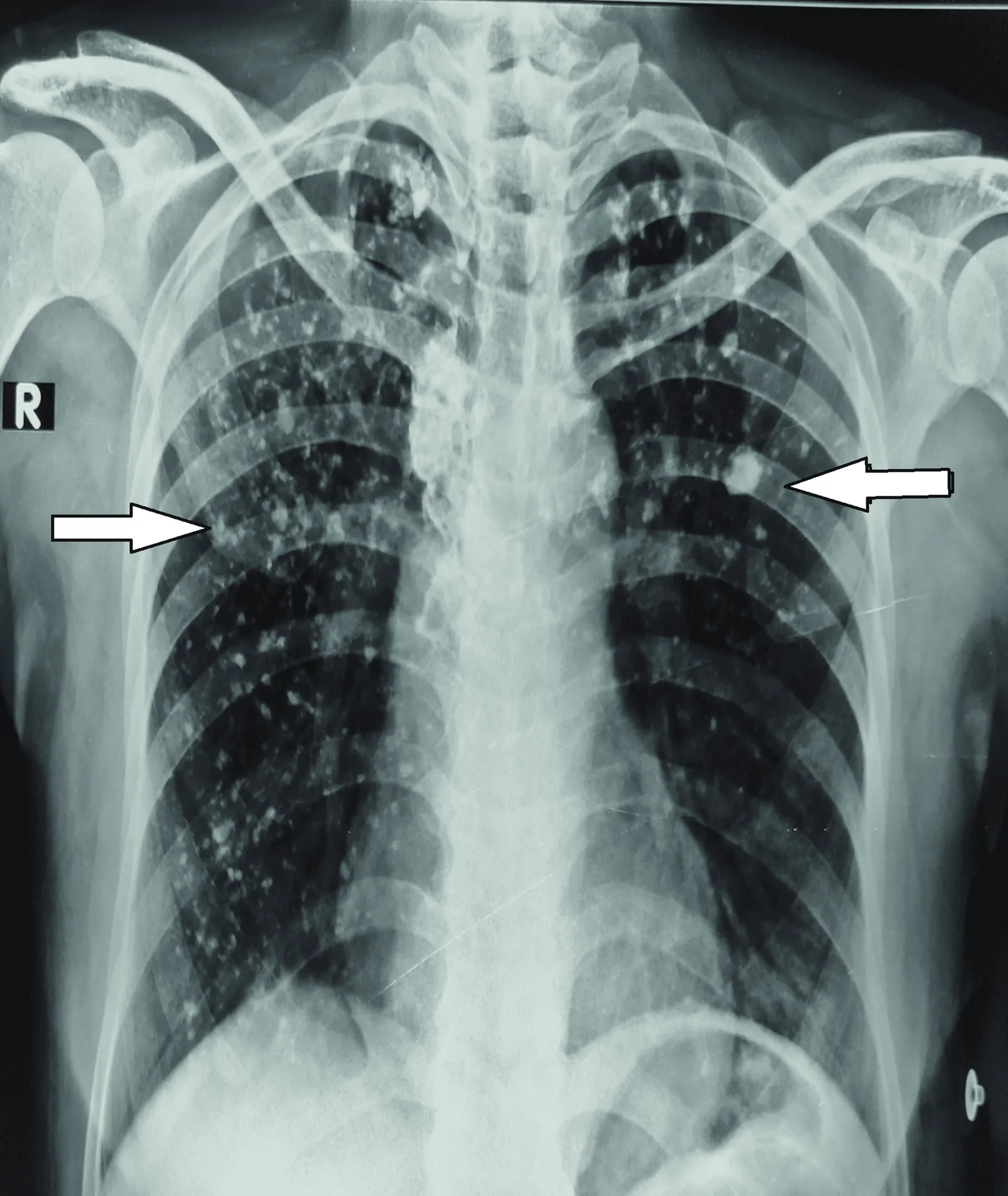
Chest X-ray showing multiple calcified pulmonary nodules Solid white arrows show the multiple healed calcified nodules suggestive of prior pulmonary tuberculosis

**Figure 4:**
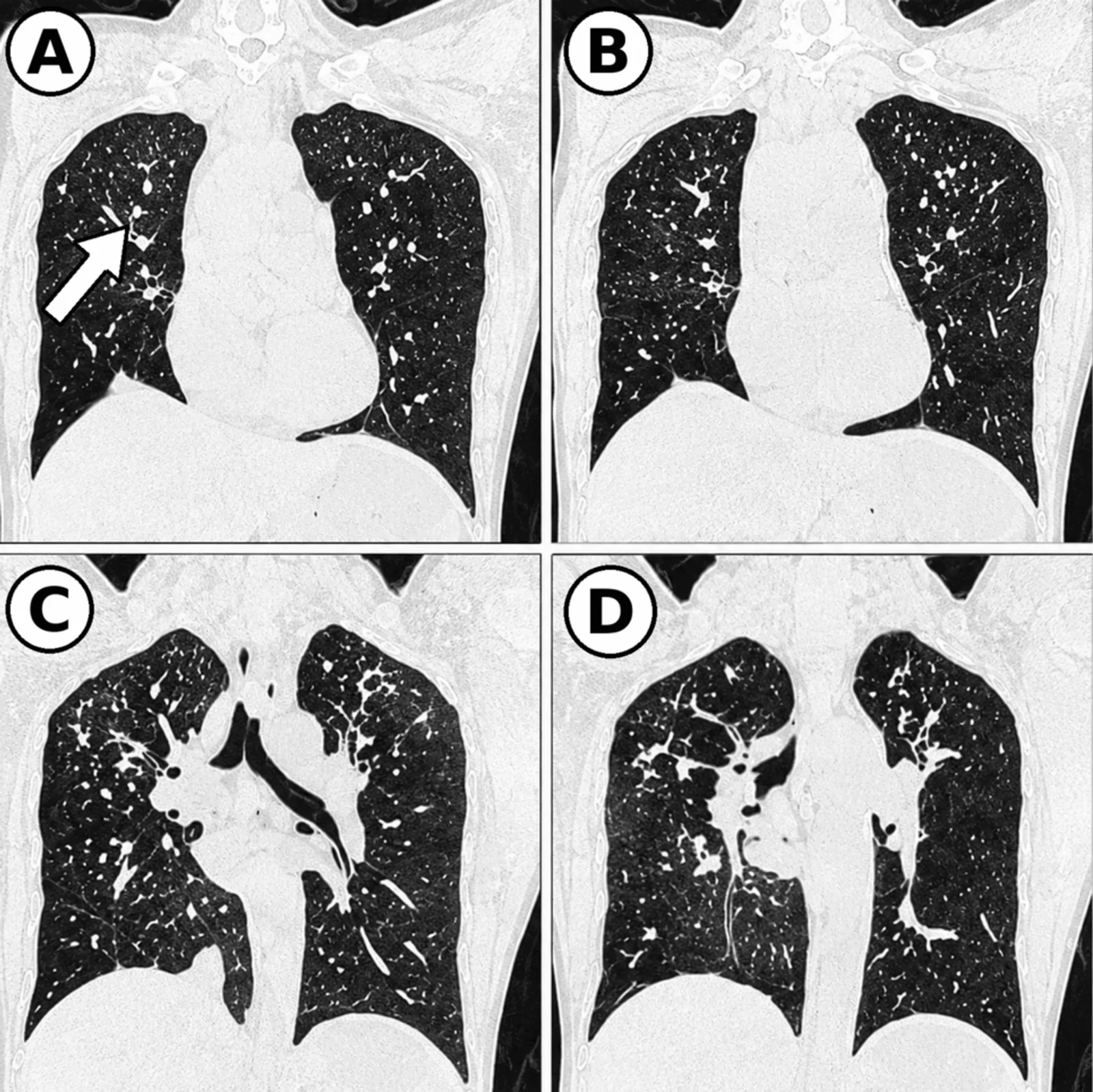
HRCT of the thorax (coronal view) showed multiple calcified pulmonary nodules Coronal HRCT image (A) showing calcified pulmonary nodules, with the solid white arrow indicating a representative calcified nodule; (B) at a subsequent level showing additional calcified pulmonary nodules; (C) showing further calcified nodules distributed through the lung parenchyma; and (D) showing the extent of calcified nodular involvement bilaterally HRCT: high-resolution computed tomography

A chest X-ray (Figure 3) and high-resolution CT (Figure 4) of the thorax showed multiple calcified pulmonary nodules suggestive of previously healed TB.

**Figure 5:**
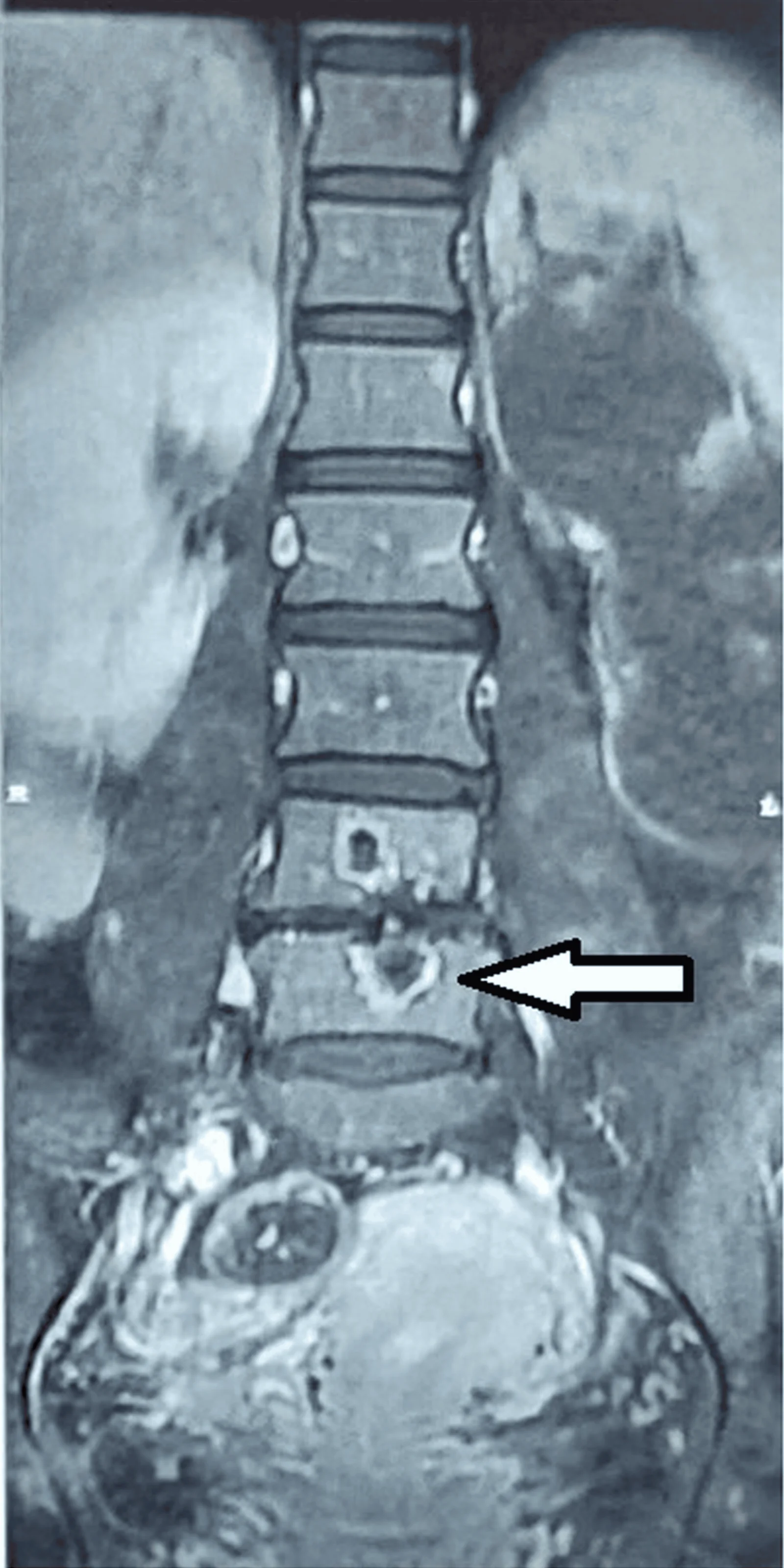
MRI LS spine (coronal view) showing multiple lytic lesions in L4-L5 vertebrae (solid arrow) MRI: magnetic resonance imaging; LS: lumbosacral

**Figure 6:**
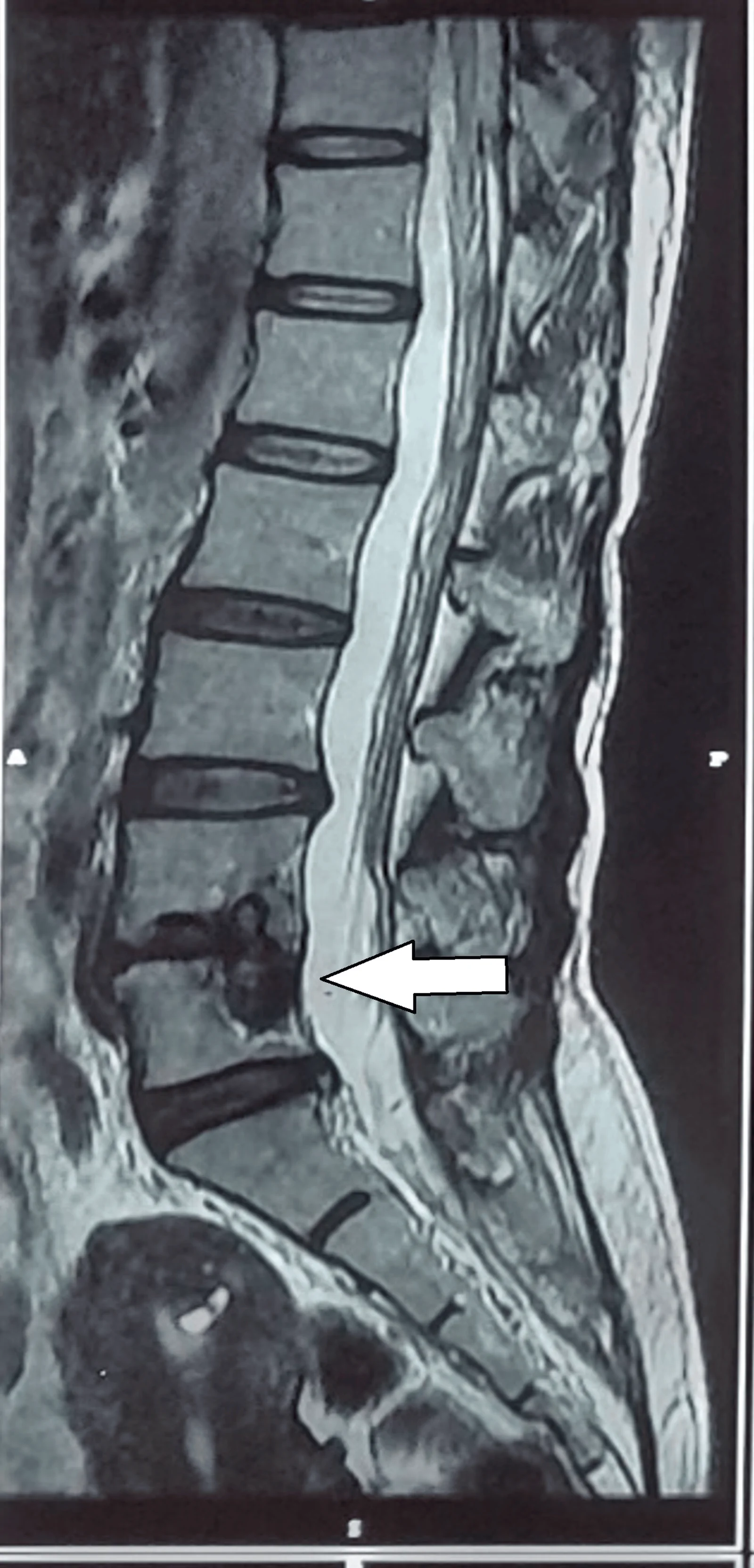
MRI LS spine (sagittal view) showing multiple lytic lesions in L4-L5 vertebrae (solid white arrow) MRI: magnetic resonance imaging; LS: lumbosacral

Magnetic resonance imaging of the spine revealed lytic lesions at the L4-L5 vertebral levels, also indicative of healed spinal TB (Pott's spine) (Figures 5, 6).

**Figure 7:**
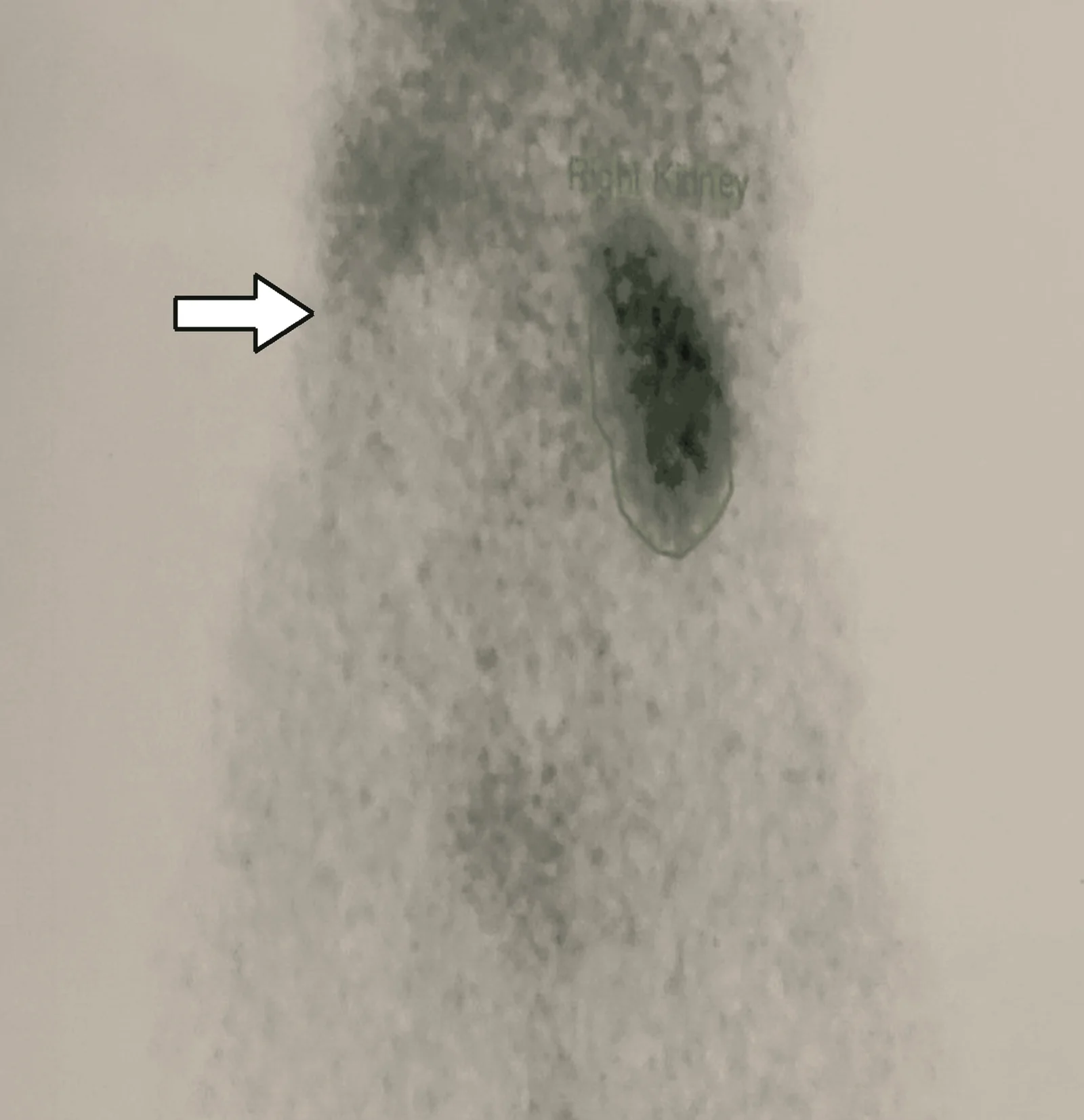
DTPA scan showing a left nonfunctional kidney (solid white arrow) DTPA: diethylenetriamine pentaacetic acid

A DTPA renal scan confirmed that the left kidney was nonfunctional (Figure 7).

**Figure 8:**
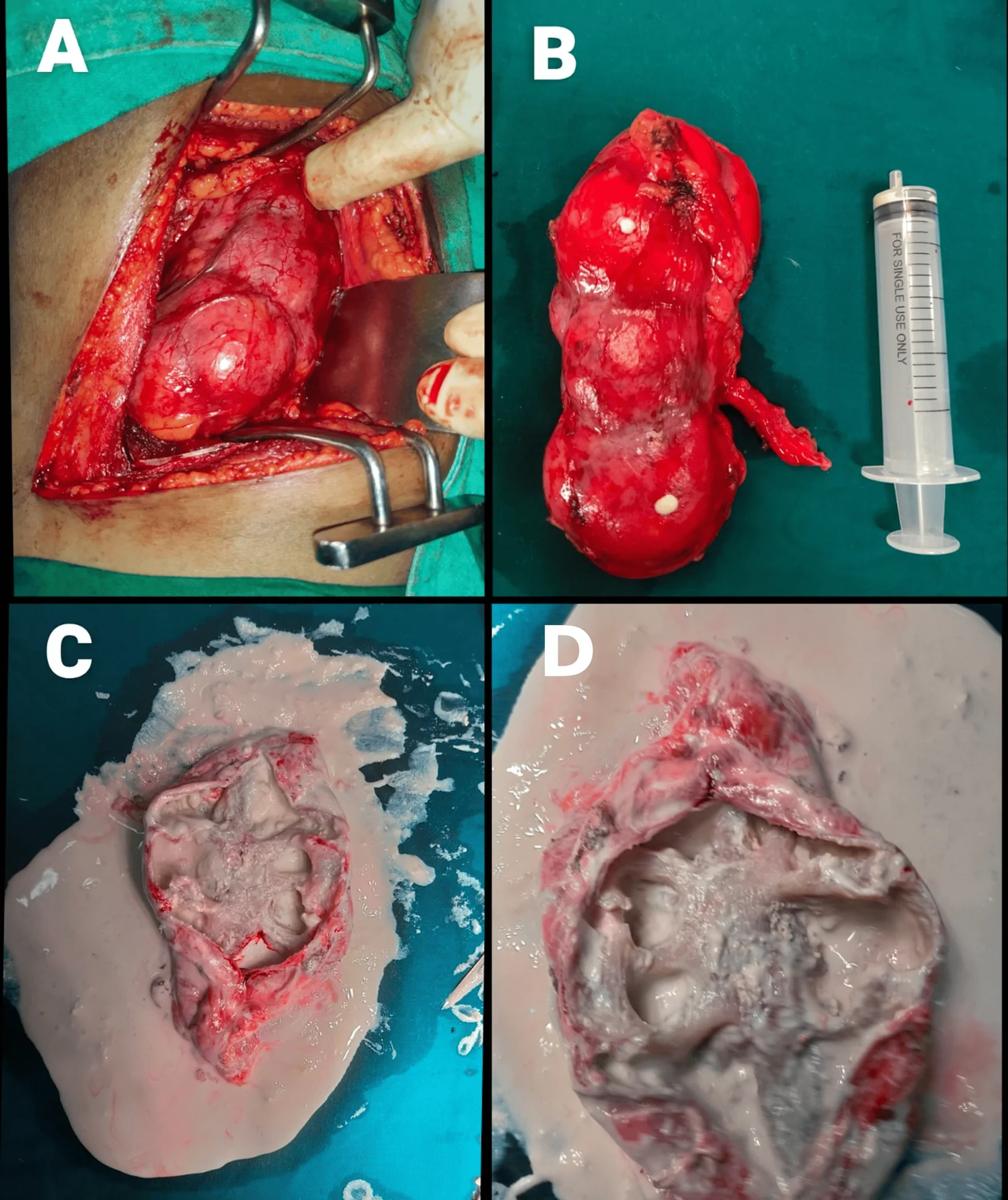
(A) Intraoperative photograph of left simple nephrectomy: exposure and mobilization of the left kidney through the 11th rib bed incision. (B) Gross specimen of nephrectomy: excised left kidney showing contracted, irregular kidney consistent with chronic renal tuberculosis. (C) Cut section of nephrectomy specimen (putty kidney): thick-walled kidney cavity filled with caseous material (putty). Extensive calcification and destruction of renal parenchyma. (D) Close-up cut section of the nephrectomy specimen showing the thick-walled cavity filled with caseous (putty) material and extensive parenchymal destruction

The patient subsequently underwent an open left simple nephrectomy through an incision along the left eleventh rib. On gross examination, the kidney was found to be largely replaced by approximately 50-60 mL of caseous necrotic material (Figure 8).

**Figure 9:**
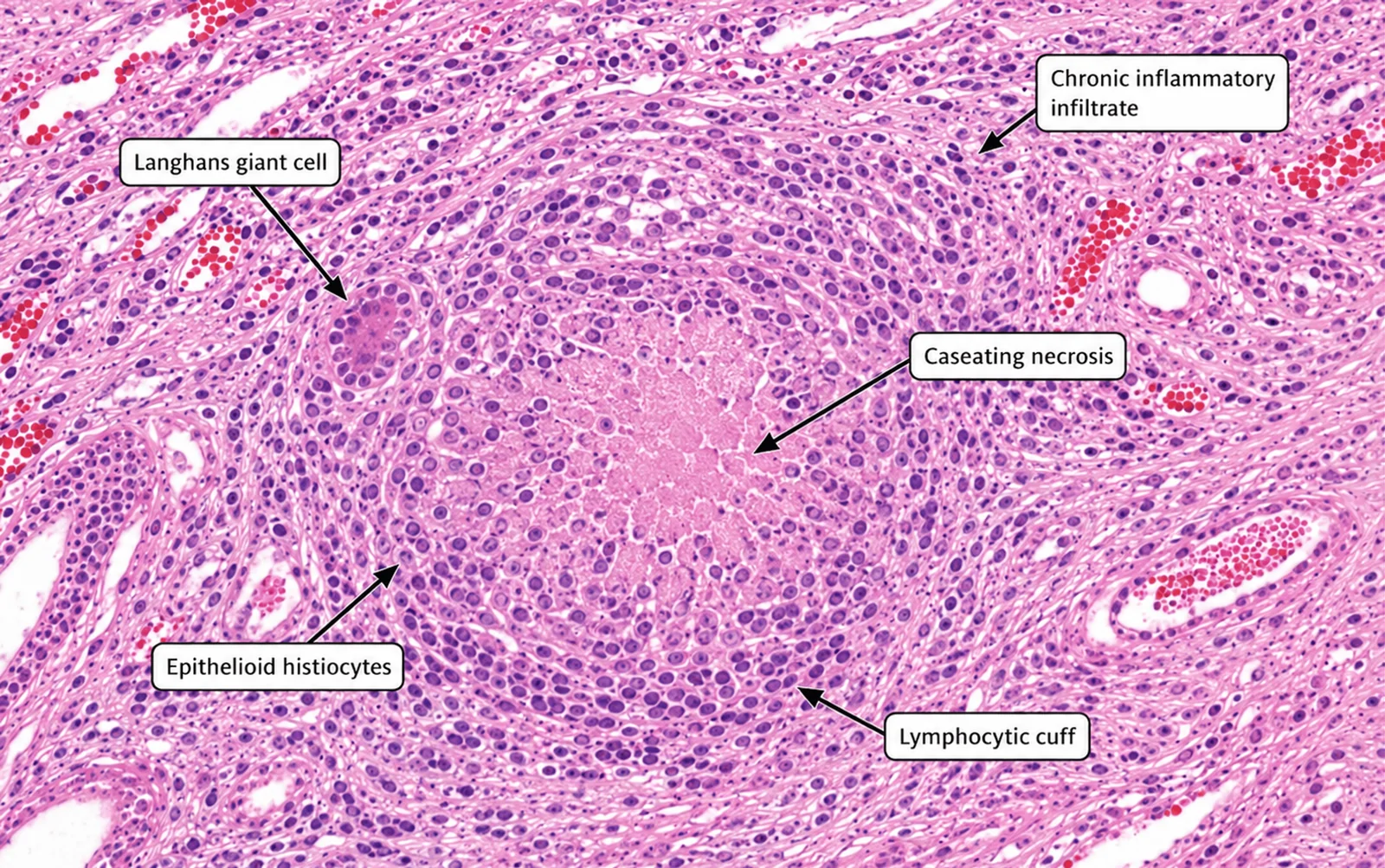
Histopathological study showing granulomatous inflammation with multinucleated giant cells and areas of caseous necrosis

Histopathological analysis demonstrated granulomatous inflammation with multinucleated giant cells and areas of caseous necrosis, confirming the diagnosis of renal TB (Figure 9).

Ziehl-Neelsen staining for acid-fast bacilli was negative. The patient’s postoperative recovery was uneventful.

## Discussion

The present case describes a 44-year-old woman with a prior history of treated pulmonary TB who was incidentally found to have a nonfunctioning putty kidney with widespread calcified granulomatous lesions involving multiple organ systems. Notably, the patient was completely asymptomatic from a urinary standpoint, emphasizing the silent and insidious progression of GUTB, even years after adequate treatment of the primary pulmonary focus.

A similar atypical presentation was reported by Qadir et al. [7], where a patient presented without classic urinary symptoms but developed acute renal failure requiring dialysis. In contrast to the present case, which was detected incidentally with preserved renal function, their case manifested with severe acute deterioration, highlighting the variable clinical spectrum of GUTB ranging from silent disease to life-threatening renal impairment.

In comparison, Mathur et al. [8] described a patient with previously diagnosed GUTB who remained noncompliant with antitubercular therapy, ultimately progressing to advanced renal failure. That patient developed extensive structural damage, including a “thimble bladder,” ureteric calcification, and a nonfunctioning kidney, with histopathology confirming a putty kidney. While both the present case and Mathur’s case demonstrate end-stage renal TB, the key difference lies in disease course: progression resulted from treatment noncompliance in their case, vs. late sequelae despite prior adequate treatment in the present case.

Similarly, Ranjan et al. [9] reported patients with prolonged symptomatic disease, including flank pain and lower urinary tract symptoms, in whom delayed diagnosis led to irreversible anatomical damage necessitating nephrectomy and reconstructive procedures. Unlike these symptomatic cases, the present patient had no urinary complaints, yet already had complete renal autodestruction, further underscoring the deceptive and subclinical nature of the disease.

Furthermore, Rocco et al. [10] demonstrated that even with timely diagnosis and standard antitubercular therapy, patients may continue to have persistent urological sequelae such as infundibular stenosis and urinary dysfunction. This finding aligns with the present case in highlighting that structural damage caused by TB is often irreversible regardless of treatment, particularly when diagnosis is delayed or the disease remains undetected for prolonged periods.

Overall, the present case is distinctive because of its entirely asymptomatic presentation, incidental diagnosis, and association with multisystem healed tuberculous sequelae many years after treated pulmonary TB. It illustrates that end-stage GUTB may remain clinically silent for years and be detected incidentally despite previous treatment for pulmonary TB. Although renal TB may become clinically apparent long after pulmonary disease, this case cannot determine whether the underlying mechanism represents reactivation of dormant bacilli or progression of previously established latent genitourinary infection. These findings support maintaining a high index of clinical suspicion and performing appropriate imaging when patients with a history of TB develop suggestive clinical or radiological findings, rather than routine imaging surveillance of all patients with previous TB.

## Conclusions

GUTB can progress insidiously and lead to irreversible renal destruction years after treated pulmonary TB. A putty kidney represents an end-stage, nonfunctioning organ that often necessitates nephrectomy. This case illustrates that end-stage GUTB may remain clinically silent for years and be detected incidentally despite previous treatment for pulmonary TB. Long-term clinical vigilance should be maintained in patients with previous TB, particularly when new symptoms or suspicious imaging findings develop. Routine imaging surveillance for all patients with previous TB is not established by current evidence.

## References

[1] Mehta PK, Kamra E (2020). Recent trends in diagnosis of urogenital tuberculosis. Future Microbiol.

[2] Roddy K, Tobin EH, Leslie SW (2024). Genitourinary Tuberculosis. https://www.ncbi.nlm.nih.gov/books/NBK557558/.

[3] Mantica G, Ambrosini F, Riccardi N (2021). Genitourinary tuberculosis: a comprehensive review of a neglected manifestation in low-endemic countries. Antibiotics (Basel).

[4] Deftereos S, Foutzitzi S (2022). The putty kidney: a classic sign from past in genitourinary radiology. Pan Afr Med J.

[5] Dumra A, Patel H (2020). Remember the “Putty Kidney” - a reminder of days gone by. Urology.

[6] Naeem M, Zulfiqar M, Siddiqui MA (2021). Imaging manifestations of genitourinary tuberculosis. Radiographics.

[7] Qadir A, Sadik N, Ghabashneh M, Almarawi H, Islam M (2025). A rare presentation of urogenital tuberculosis leading to obstructive uropathy and renal failure: a case report. BMC Urol.

[8] Mathur M, Kayal A, Beniwal P, Agarwal D, Malhotra V (2014). A neglected case of genito-urinary tuberculosis; presenting with “putty” kidney and “thimble” bladder. South East Asian J.

[9] Ranjan SK, Mittal A, Kumar S, Panwar VK, Mammen KJ, Usha P (2019). “Putty” kidney with “thimble” bladder, a disastrous sequela of neglected genitourinary tuberculosis: a call for surgical intervention. Int J Sci Res.

[10] Rocco R, Villar BB, da Cunha Ferreira L (2025). Renal tuberculosis with genitourinary sequelae: a case report. Rev Inst Med Trop Sao Paulo.

